# The Effect of Commercially Available Endodontic Cements and Biomaterials on Osteogenic Differentiation of Dental Pulp Pluripotent-Like Stem Cells

**DOI:** 10.3390/dj6040048

**Published:** 2018-09-22

**Authors:** Atari Maher, Raquel Núñez-Toldrà, Neus Carrio, Eduard Ferres-Padro, Hamad Ali, Sheyla Montori, Ashraf Al Madhoun

**Affiliations:** 1 UIC Regenerative Medicine Research Institute, Universitat Internacional de Catalunya, St Josep Trueta s/n, Sant Cugat del Vallès, 08195 Barcelona, Spain; rnunez@uic.es (R.N.-T.); neuscarrio@yahoo.es (N.C.); smontori@uic.es (S.M.); 2 Oral and Maxillofacial Surgery Department, Fundació Hospital de Nens de Barcelona, 08017 Barcelona, Spain; eduard.fp@institutferresamat.com; 3 Department of Medical Laboratory Sciences (MLS), Faculty of Allied Health Sciences, Health Sciences Center, Kuwait University, 90805 Sulaibikhat, Kuwait; hamad.ali@HSC.EDU.KW; 4 Functional Genomics Unit, Research Division, Dasman Diabetes Institute, 15462 Dasman, Kuwait

**Keywords:** dental pulp pluripotent-like stem cell, dental pulp, pluripotency, Biodentine, MTA, Med-PZ, osteogenic differentiation, biomaterials, osteogenesis

## Abstract

The aim of this study is to compare the osteogenic differentiation capacity of the dental pulp pluripotent-like stem cells (DPPSCs) using conditional media pretreated with ProRoot-MTA, Biodentine (BD) or the newly manufactured pure Portland cement Med-PZ (MZ). DPPSCs, isolated from human third molars, are the most relevant cell model to draw conclusions about the role of biomaterials on dental tissue regeneration. Cytotoxicity, alkaline phosphatase (ALP) activity, and calcium deposition analysis were evaluated at different differentiation time points. Gene expression of key osteogenic markers (*RUNX2*, Collagen I and Osteocalcin) was determined by qRT-PCR analysis. The osteogenic capacity of cells cultured in conditioned media prepared from MZ or MTA cements was comparable. BD conditioned media supported cell proliferation but failed to induce osteogenesis. Relative to controls and other cements, high osteogenic gene expression was observed in cultures pre-treated with the novel endodontic cement MZ. In conclusion, the in vitro behavior of a MZ- endodontic cement was evaluated, showing similar enhanced cell proliferation compared to other commercially available cements but with an enhanced osteogenic capacity with prospective potential as a novel cement for endodontic treatments.

## 1. Introduction

Since the introduction of mineral trioxide aggregate (MTA) into the field of endodontics, calcium silicate-based materials, also known as bio-ceramic cements, have gained recognition for their biocompatibility, antibacterial capacity and regenerative properties [1,2]. MTA have been extensively used in clinical applications including, apical barriers, root perforations repairs, root-end fillings, direct pulp capping and pulpotomies [3,4]. The ProRoot MTA from Dentsply (Tulsa, OK, USA) is primarily made of tricalcium and di-calcium silicate salts [5]. MTA has been shown to upregulate the differentiation of osteoblasts, fibroblasts, cementoblasts, odontoblasts and pulp cells [6,7]. However, MTA is difficult to handle, labor and cost intensive. To overcome these limitations, two calcium-silicate based biomaterials have been recently introduced to the market. Biodentine (BD) and pure Portland cement Med-PZ (MZ) from Septodont (St. Maur des Fosses, France) and Medcem GmbH (Weinfelden, Switzerland), respectively. The manufacturers of BD claim beneficial effects of their products including low gingival fibroblast cytotoxicity [8]. It has been successfully used as a coronal restorative material for perforation repair and pulp-capping [9]. On the other hand, MZ is composed of the raw materials, similar to MTA, but with significant reduced cost [10]. Recent reports indicate that tricalcium silicate, the main component in both cements, is bioactive, degradable and self-setting bone filling material [10,11,12].

Dental pulp tissue is a promising source of stem cells, which are widely used to test biomaterials, reviewed in [13,14]. In this context, we recently established a protocol for isolating and identifying a new subpopulation of pluripotent-like stem cells from dental pulp, named Dental Pulp Pluripotent-like Stem Cells (DPPSCs) [15], that express pluripotent markers and show genetic stability [16,17]. In vitro studies revealed that DPPSCs form embryoid body-like structures (EBs) and may generate the three embryonic germ layers [18]. Thus, DPPSCs can differentiate into tissues that have similar characteristics to mesoderm, endoderm and ectoderm layers. In this study, we evaluated the cytotoxic effect and the osteogenic capacity of DPPSCs cultured in conditional pre-treated with the three biomaterials MZ, BD, or MTA individually. 

## 2. Materials and Methods

### 2.1. Patient Selection

The third molars of three healthy humans were extracted from 14–20 years old female and male donors for orthodontic and prophylactic reasons as previously described [15]. Gentle extraction procedures were used to preserve teeth from damage. For experimental purposes, the dental pulp tissues were extracted from the teeth with the patient consent [16,17]. 

All subjects gave their informed consent for inclusion before they participated in the study. The study was conducted in accordance with the Declaration of Helsinki. The protocol approval and the guidelines on human stem cell research was issued by the Committee on Bioethics of the *Universitat Internacional de Catalunya* (study code BIO-ELB-2013-03).

### 2.2. Biomaterials Preparation

Plastic ring discs (16 mm^3^) were prepared, disinfected with ethanol and sterilized by plasma [19]. Each of the dental biomaterials: Biodentine^TM^ (BD, Septodont, St. Maur des Fosses, France), ProRoot MTA^®^ (MTA, Dentsply, Tulsa, OK, USA) and Pure Portland cement^®^ MZ (MZ, Medcem GmbH, Weinfelden, Switzerland) was individually prepared in accordance with the manufacturer’s instructions, under aseptic conditions. Plastic ring discs were pre-treated with each of the prepared biomaterial individually in triplicate with total of nine rings (*n* = 9). After setting, the biomaterial-disc samples were placed in 24-well plates and incubated for 24 h at 37 °C, 5% CO_2_ atmosphere and at 98% humidity [20]. The samples were subsequently expose to ultraviolet light for 20 min and placed in 2 mL of basal medium (DMEM-low glucose (Life Technologies, Carlsbad, CA, USA), penicillin (100 units/mL) and streptomycin (100 µg/mL), 10% fetal bovine serum (FBS; Sigma, St. Louis, MO, USA), Control samples were prepared from discs with no cements containing 400 µL of basal media; as described by Zanini et al. [21,22]. After 24 h, the conditioned medium soaked with the biomaterials was individually filtered through 0.22 µm filters and then used for further experiments.

### 2.3. Isolation and Culture of Human Dental Pulp Pluripotent-Like Stem Cells (DPPSCs)

Human DPPSCs were isolated and cultured as previously reported [16]. Cells at passage 12 were cultured in DPPSC maintenance medium, consisting of 60% DMEM-low glucose (Life Technologies, Carlsbad, CA, USA) and 40% MCDB-201 (Sigma, St. Louis, MO, USA); supplemented with 1× insulin-transferrin-selenium (ITS; Sigma, St. Louis, MO, USA), 1× linoleic acid-bovine serum albumin (LA-BSA; Sigma, St. Louis, MO, USA), 1 nM dexamethasone (Sigma, St. Louis, MO, USA), 0.1 mM ascorbic acid 2-phosphate (Sigma, St. Louis, MO, USA), penicillin (100 units/mL) and streptomycin (100 µg/mL), 2% fetal bovine serum (FBS; Sigma, St. Louis, MO, USA), 10 ng/mL hPDGF-BB (R&D Systems, Minneapolis, HN, USA) and 10 ng/ml EGF (R&D Systems, Minneapolis, HN, USA). Cell passage was performed every 4 days. Cells from passage 14 were seeded in 24-well plates at a density of 4 × 10^3^ cells/well. Cells were cultured overnight to allow complete adhesion [15].

### 2.4. Osteogenic Differentiation

DPPSCs were seeded in the conditioned media (BD, MZ, MTZ or control), upon 80% confluency; cells were treated with the osteogenic medium containing DMEM-low glucose supplemented with 10% FBS, penicillin (100 units/mL), streptomycin (100 µg/mL), 100 nM dexamethasone, 10 mM µ-glycerophosphate and 50 µg/mL ascorbate-2-phosphate. The Culture medium was replaced every 3 days for 10 days. Cells were fixed, and osteoblasts were detected using Alkaline phosphatase detection Kit (Millipore, Burlington, MA, USA) following the manufacturer protocol. Control cells were gown in culture medium in the absence of the biomaterials.

### 2.5. Cell Proliferation Assay

The DPPSCs were cultured on conditioned media (BD, MZ or MTZ) or basal media for 3, 7 and 10 days. The cellular proliferation was measured using 3-(4,5-dimethylthiazol-2-yl)-2,5-diphenyl tetrazolium bromide (MTT) assay; following the manufacturer’s instructions (Tebu-Bio, Barcelona, Spain). The optical densities of the viable cells were measured at wavelength 570 nm and compared to the reference wavelength 630 nm on a multi-plate reader (Unicam Helios alfa). 

### 2.6. Alkaline Phosphatase Activity (ALP) Analysis

A total number of 4 × 10^3^ DPPSCs were cultured in each well of 24-well plates with the corresponding pre-conditioned media (BD, MZ, MTZ or control). At days 7 and 10, the ALP Staining kit (CosmoBio, Carlsbad, CA, USA) was used in accordance with the manufacturer’s instructions. ALP- staining was observed and cell images were taken on optical microscope (Euromex, Arnhem, The Netherlands). Alkaline phosphatase activity was quantified by spectrophotometry and the absorbance was measured at 405 nm for a period of 1 and 2 min. 

### 2.7. Calcium Accumulation

Calcium cellular content was evaluated using Sigma Kit (Sigma, St. Louis, MO, USA). In accordance with the manufactures protocol, differentiated DPPSCs cultured on BD-, MZ-, MTZ- or control- conditional media were washed twice with 1x PBS and incubated in lysis solution. The total cellular calcium content was measured using the standard solutions, provided with the Kit and the interaction absorbance was measured at 575 nm. In addition, calcium cell deposits were detected with Von Kossa staining. 

### 2.8. RNA Extraction, cDNA Synthesis and qRT-PCR Reactions

At day 10 post-differentiation, Trizol protocol (Invitrogen, Carlsbad, CA, USA) was used to extract total RNAs from the differentiated cells- seeded in the previously described conditioned media. M-MLV Reserve Transcriptase Kit (Invitrogen, Carlsbad, CA, USA) was used to prepare cDNA from 2 µg total RNA. qRT-PCR assays were performed as previously described [23,24]. Primer pairs with equivalent efficiencies are described in Table 1. qRT-PCR assays were performed using CFX96 system (Bio-Rad, Hercules, CA, USA) as previously described [25,26]. The results were normalized to the GAPDH- CT-values, and averages ± SEM are shown expressed relative to Control or Day 0 undifferentiated cells, as indicated. 

### 2.9. Statistical Analysis

All data were analyzed at the Statistical Department at the Universitat Internacional de Catalunya. ANOVA of two factors and the Kruskal Wallis test, with statistical significance set at *p* < 0.05 were applied to compare cell populations. The values were expressed as the Mean ± Standard Deviation. Statgraphics^®^ Centurion (StatPoint Technologies, Inc., Warrenton, VA, USA) was used for data analyses.

## 3. Results

### 3.1. Cellular Morphology and Viability

**Figure 1:**
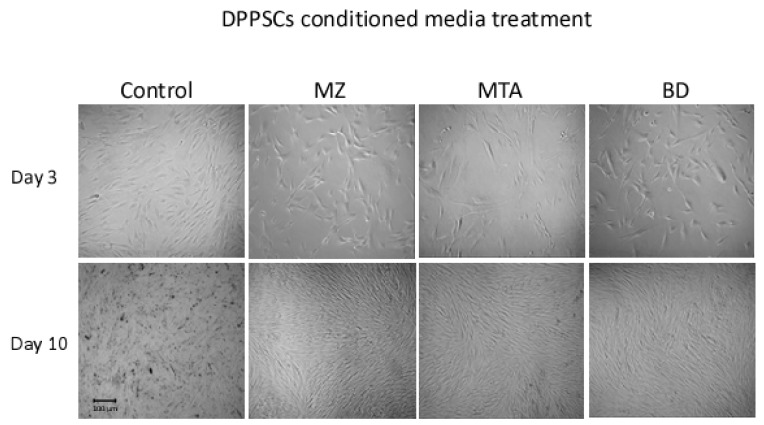
Cell morphology. Dental Pulp Pluripotent-like Stem Cells (DPPSCs) at day 3 (100×) and at day 10 (40×) of differentiation. BD, Biodentine; MZ, MedCem; MTA, Mineral Trioxide Aggregate; CT, Control media.

Light microscope image observations revealed a stable cellular proliferation from day 3 to day 10 in all tested groups. Relative to control basal culture media, at differentiation day 3, DPPSCs cellular morphology was similar independent from the source of the conditional media (Figure 1). At day 10, the death rate of cells cultured under control conditions was observed. In contrast, cells grown in BD-, MZ- or MTA-conditioned media were steady and showed elongated morphology, relative to day 3 (Figure 1).

**Figure 2:**
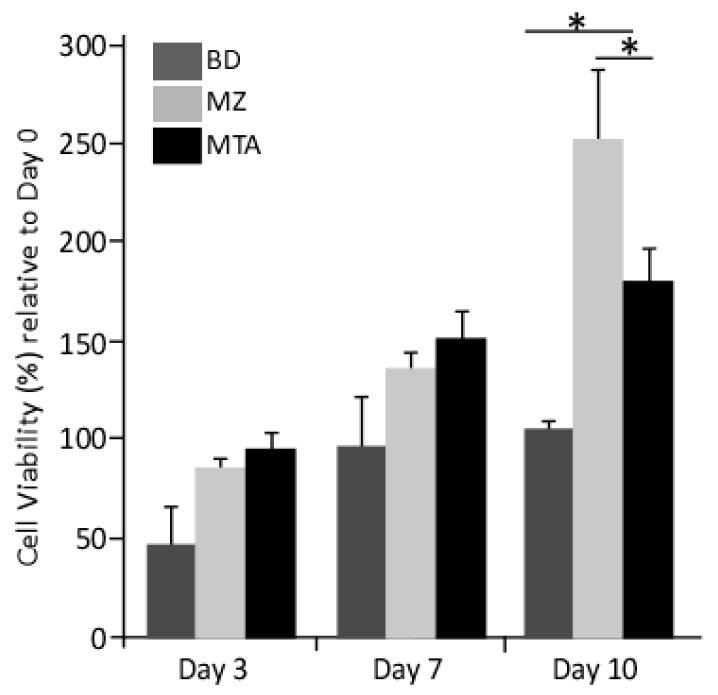
Proliferation MTT assay. The data were represented as the average ± SD (*n* = 3) versus cells cultured on control media. Average of the MTT absorbance in the 3 experimental groups and the control. There are significance differences between BD and MZ and between BD and MTA. (*: *p* < 0.01).

In the presence of conditioned media, we observed an improvement in DPPSCs cell viability (Figure 2) relative to that observed in cells cultured on basal media. The cellular viability was enhanced two-three fold in the presence of MZ- or MTA-conditioned media at days 3 and 7. Notably, at day 10, the proliferation rate of cells cultured in MZ-conditioned media was 2.5- and 1.5-fold relative to that of cells cultured on MTA- or BD-conditioned media, respectively (Figure 2). 

### 3.2. Osteogenic Differentiation

**Figure 3:**
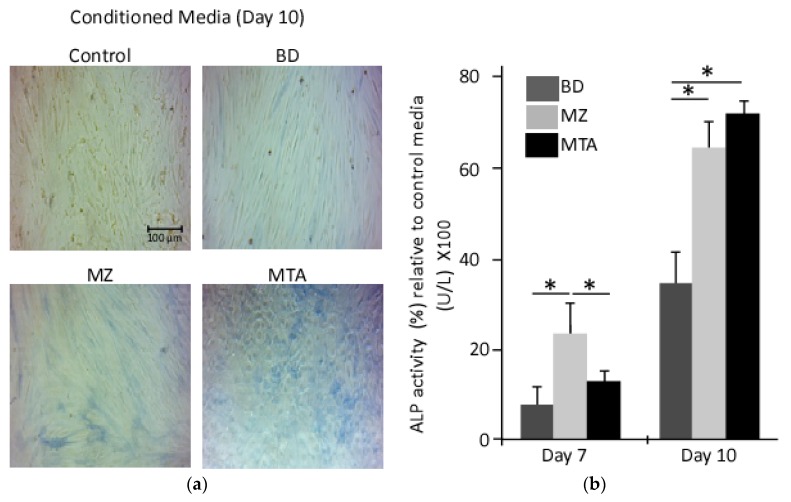
Alkaline phosphatase activity assay. A time course detection for ALP activity in differentiated DPPSCs seeded on conditioned media, days 7 and 10. (**a**) ALP was detected with the chromogenic substrate from the ALP staining kit; (**b**) ALP activity was measured in differentiated DPPSCs seeded on the biomaterials conditional media, days 7 and 10 (*: *p* < 0.01).

In the presence or absence of the biomaterials, the osteogenic differentiation of DPPSCs was determined by culturing them in media containing dexamethasone, µ-glycerophosphate and ascorbate as described in the Material and Methods section. Except for the control conditioned media, on day 10 of differentiation, cells showed remarkable changes in cellular morphology and stained positive for alkaline phosphatase activity (Figure 3a). In the presence of the biomaterial conditioned media, spectrophotometry analysis revealed a significant induction of alkaline phosphatase activity relative to that observed in control conditioned at days 7 and 10 during osteogenesis. Interestingly, DPPSCs cultured on MZ- or MTA-conditioned media showed comparable ALP activity with 2-fold higher than cells differentiated in the presence of BD conditions (differentiation day 10, Figure 3b).

**Figure 4:**
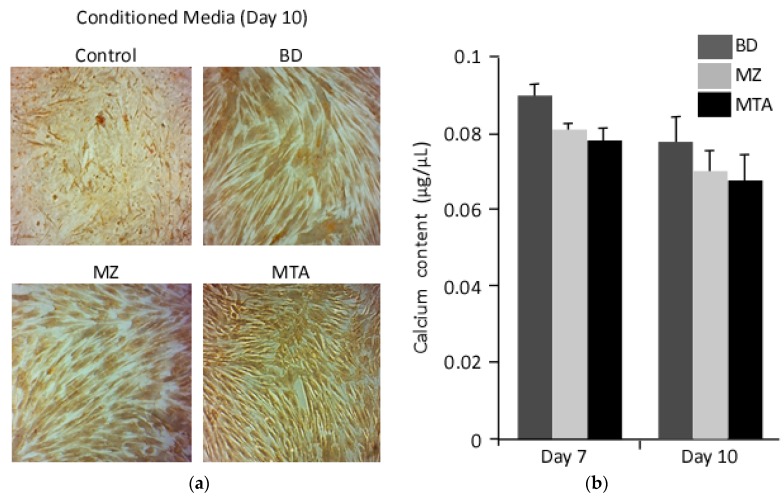
Calcium composition. (**a**) Calcium deposits were detected with Von Kossa at day 10. (**b**) Calcium cell composition was measured in differentiated DPPSCs seeded on the biomaterials conditional media, days 7 and 10.

Similarly, microscopic images for calcium deposits revealed a pronounce staining of DPPSCs differentiated in the presence of MZ- or MTA-conditioned media (Figure 4a). However, calcium content measurements revealed no significant differences between cells differentiated in the presence of the biomaterials used in this study.

**Figure 5:**
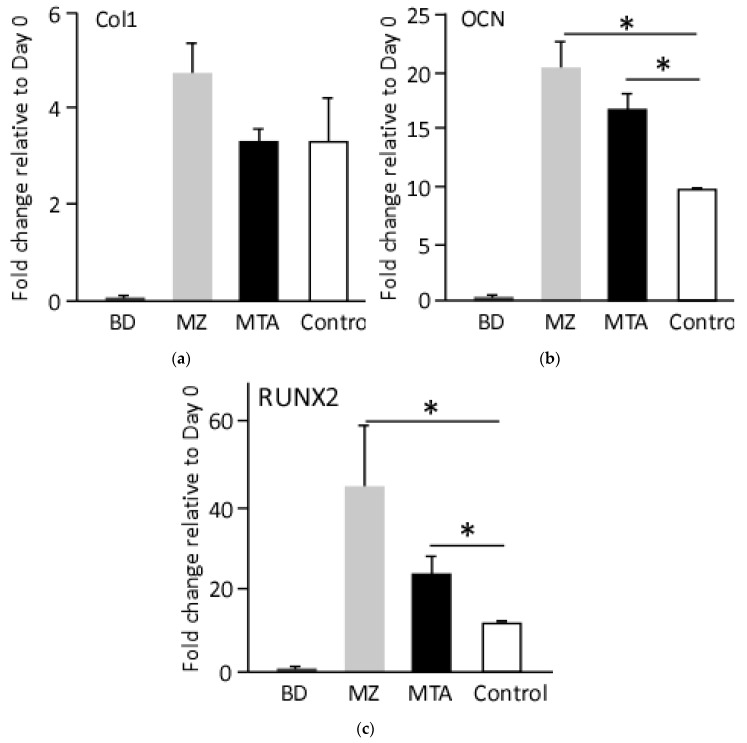
Molecular evaluation of the generated osteoblasts. qRT-PCR analysis for the expression of osteogenic markers, at day 10 post-differentiation. Values were normalized to the housekeeping gene *GADPH* and undifferentiated cell. (**a**) Col1; (**b**) OCN; (**c**) RUNX2, (* *p* < 0.01).

At molecular level, qRT-PCR analysis, at day 10 of osteogenic differentiation, revealed an upregulation of the osteogenic genes collagen I (Col1), osteocalcin (OCN) and Runt-related transcription factor 1 (RUNX1) relative to day 0 and control cells. In comparison to undifferentiated cells, cell treated with the basic control media showed 3.5-fold increase in Col1 transcripts, whereas, cell treatments with MZ- or MTA-conditioned media resulted in 5- and 3-fold induction of Col1, respectively (Figure 5). On the other hand, OCN transcripts were comparably up-regulated in cells treated with MZ- or MTA-conditioned media, 2- and 1.7-fold, relative to cells differentiated in the basic media, respectively. Similarly, the expression of osteogenic transaction factor, RUNX2, was the highest in cells treated with MZ-conditioned media (5-fold) and MTA-conditioned media (2.2-fold) relative to control cells (Figure 5). Interestingly, BD-conditioned media did not enhance the expression of any osteogenic gene markers, suggesting failure of this media to mediate osteogenesis (Figure 5).

## 4. Discussion

The in vitro assays to analyze the toxicity and functionality of endodontic materials are highly important for the enhancement of root canal therapy. Yet, current studies mostly use non-dental cells, such as fibroblast cells or cancer cell lines [20], which may not be an ideal cell model to draw conclusions about the usage of biomaterials on dental tissue. In the present study, DPPSCs isolated from a healthy human third molar were used because they represent an easily accessible cell source with pluripotent phenotype and genetic stability [15,17,27].

Cells have been demonstrated to behave differently when cultured on the same material surface. Differences were observed between the periodontal ligament (PDL) fibroblasts and the mouse fibroblast cell line (L929) when measuring toxicity of dental materials [28]. The PDL fibroblast cells are less sensitive than L929 cells, and ultrastructural differences between the two cell types were determined. Similarly, different cell types, e.g., fibroblasts and osteoblasts, respond contrarily to a wide range of biomaterials [29]. In the current light of tissue engineering, it is recommended to use stem cell isolated from dental tissue as ideal source for conducting research on the efficiency of biomaterial for prospective clinical application [30]. Hence, we used a new source of stem cells, DPPSCs isolated from dental pulp tissue as an alternative cell model to evaluate biomaterials.

The functional role of MTA and BD have been well demonstrated in several studied [22,31]; however, to our knowledge, there are no published reports on the in vitro usage of MZ-biomaterials. Recent applications for regenerative therapies require bioactive dental materials to induce tissue regeneration [20]. It has been demonstrated that calcium silicate Portland-derived cements (like MTA or Med-PZ) allow the growth and differentiation of dental pulp cells [32], osteoblasts [33], human orofacial mesenchymal stem cells [34] and cementoblasts [35]. Therefore, the main objective of this study was to compare the osteogenic differentiation capacity DPPSCs cultured in conditional media pre-treated with MZ, MTA or BD cements.

In our study, we observed that cell proliferation differences exist in response to the different treatments. Unlike BD-conditioned media, cytotoxicity tests revealed that both MZ- and MTA-conditioned media maintain cell viability and proliferation. This effect could be due to the high concentration of Biodentine that was used in our study. Luo et al. recommended the use of low concentration of BD-biomaterial in the range of 0.2 mg/mL [36], thus, the concentration used in the present study could had a negative effect on the cells. Our data are in correlation with the findings of Pérard et al. who studied MTA- and BD-conditional media on two different odontoblast-like cell types (MDPC-23 and Od-21 cells) [19]. They reported that MTA-pre-treated medium induces cellular proliferation, in comparison to BD-control medium. On the other hand, Attik et al. have used MG63 human osteoblast-like cells (osteosarcoma cells) to compare Biodentine™ and ProRoot^®^ MTA. They reported similar cytotoxic effects of both materials, but the MG-63 carcinogenic cell line may have a different response to biomaterials [33].

ALP is a marker for an early onset of osteogenesis. The protein expression and its kinetic activity are notably elevated during stem cell differentiation into osteogenic lineage. Furthermore, during the bone turnover, ALP mediate the degradation of inorganic pyrophosphate and the release of phosphate groups for minimization process [34]. ALP activity, which is closely related to new bone formation, was used as a measure of osteo-conductivity of the tested materials [37]. Peng et al. demonstrated that tricalcium silicate cement had a higher ALP activity compared to the controls at day 10, using hDPCs [38]. In our study, ALP activity was increased over days in MTA- and MZ-groups as compared with the BD-group. Gypsum or calcium sulfate, the main component of MTA and MedCem, has many applications in medicine and dentistry [36]. It is a fast-degradable material that usually resorbs before the onset of the new bone regeneration. Dewi et al. in an in vitro and in vivo study, concluded that the addition of CaCO_3_ hydrogel into calcium sulfate increases bone formation, angiogenesis and collagen density and results in a faster bone formation and maturation [39].

At molecular level, the expression of three osteogenic markers in differentiated DPPSCs cultured in three conditioned media showed that Col1, an early osteoblast marker, is highly up-regulated in cells differentiated in the presence of MTA- or MZ-, relative to BD-, conditioned media. Runx2, an essential transcription factor for the onset of osteoblast, is dramatically up-regulated in cells treated with MZ-conditioned media, as compare to its expression in the other groups. Similarly, as an indicative of mineralization phase entry, MZ-conditioned treatment enhanced the expression of OCN, a non-collagenous protein found in bone, dentin and osteoblasts [40].

These results may be explained by the fact that cells cultured in MTA- or MZ-conditioned media could mediate DPPSCs’ osteogenesis. Eid et al. studied different types of tricalcium silicate cements on human bone marrow-derived mesenchymal stem cells in vitro, but they observed a downregulation of RUNX2 and OCN transcripts [41]. In accordance with our study, Zanini et al. observed a downregulation of RUNX2 on OD-21 cells (mouse undifferentiated pulp cells) which cultured in 1 mg/mL BD [21]. In contrast, Peng et al. reported an up-regulation of Col1 and OCN in cells cultured in the presence of tricalcium silicate cement [38]. Thus, the differences in the osteogenic genes expression levels can be explained by the differences in the cell origin and the developmental status at the time incubated with the conditioned media. 

## 5. Conclusions

In light of the results, we conclude that there are differences between BD, MTA and MZ in terms of cellular cytotoxicity, the onset of osteogenic differentiation of DPPSCs, cellular mineralization and the expression of osteogenic markers in differentiated cells. Unlike MTA and MZ, conditioned media from BD has no capacity to mediate osteogenesis in DPPSCs. 

## Figures and Tables

**Table 1:** Oligonucleotide sequences of primers utilized for real-time qRT-PCR.

Genes	Forward Primer (5′-3′)	Reverse Primer (5′-3′)
GAPDH	GGAGCGAGATCCCTCCAAAAT	GGCTGTTGTCATACTTCTCATGG
Col1	TGCTGGCAAGAATGGCGATC	CTGTCTCAGCCTTGTCACCAC
BMP2	TGGTTACTGTCATGGCGGGTA	TCTCAGATCGTTGAACCTTGCTA
OCN	CTCACACTCCTCGCCCTATT	GCTCCCAGCCATTGATACAG

## Abbreviations

BD	Biodentine™
MZ	Pure Portland cement^®^ Med-PZ
MTA	ProRoot^®^ Mineral Trioxide Aggregate
DPSCs	Dental Pulp Stem Cells
DPPSCs	Dental Pulp Pluripotent-like Stem Cells
MTT	3-(4,5-dimethylthiazol-2-yl)-2,5-diphenyl tetrazolium bromide
ALP	Alkaline Phosphatase
RUNX2	Runt-related transcription factor 2
COL1	Collagen type I
OCN	Osteocalcin
GAPDH	glyceraldehyde-3-phosphate dehydrogenase

## References

[1] Parirokh M., Torabinejad M. (2010). Mineral trioxide aggregate: A comprehensive literature review—Part I: Chemical, physical, and antibacterial properties. J. Endod..

[2] Torabinejad M., Parirokh M. (2010). Mineral trioxide aggregate: A comprehensive literature review—Part II: Leakage and biocompatibility investigations. J. Endod..

[3] Perez A.L., Spears R., Gutmann J.L., Opperman L.A. (2003). Osteoblasts and MG-63 osteosarcoma cells behave differently when in contact with ProRoot MTA and White MTA. Int. Endod. J..

[4] Parirokh M., Torabinejad M. (2010). Mineral trioxide aggregate: A comprehensive literature review--Part III: Clinical applications, drawbacks, and mechanism of action. J. Endod..

[5] Camilleri J., Montesin F.E., Brady K., Sweeney R., Curtis R.V., Ford T.R. (2005). The constitution of mineral trioxide aggregate. Dent. Mater..

[6] Hakki S.S., Bozkurt S.B., Hakki E.E., Belli S. (2009). Effects of mineral trioxide aggregate on cell survival, gene expression associated with mineralized tissues, and biomineralization of cementoblasts. J. Endod..

[7] Okiji T., Yoshiba K. (2009). Reparative dentinogenesis induced by mineral trioxide aggregate: A review from the biological and physicochemical points of view. Int. J. Dent..

[8] Zhou H.M., Shen Y., Wang Z.J., Li L., Zheng Y.F., Häkkinen L., Haapasalo M. (2013). In vitro cytotoxicity evaluation of a novel root repair material. J. Endod..

[9] Laurent P., Camps J., De Meo M., Dejou J., About I. (2008). Induction of specific cell responses to a Ca(3)SiO(5)-based posterior restorative material. Dent. Mater..

[10] Huan Z., Chang J. (2007). Self-setting properties and in vitro bioactivity of calcium sulfate hemihydrate-tricalcium silicate composite bone cements. Acta Biomater..

[11] Zhao W.Y., Chang J. (2004). Sol-gel synthesis and in vitro bioactivity of tricalcium silicate powders. Mater. Lett..

[12] Zhao W.Y., Wang J., Zhai W.Y., Wang Z., Chang J. (2005). The self-setting properties and in vitro bioactivity of tricalcium silicate. Biomaterials.

[13] Horst O.V., Chavez M.G., Jheon A.H., Desai T., Klein O.D. (2012). Stem cell and biomaterials research in dental tissue engineering and regeneration. Dent. Clin. N. Am..

[14] Sun J.X., Horst O.V., Bumgarner R., Lakely B., Somerman M.J., Zhang H. (2012). Laser capture microdissection enables cellular and molecular studies of tooth root development. Int. J. Oral Sci..

[15] Atari M., Barajas M., Hernández-Alfaro F., Gil C., Fabregat M., Ferrés Padró E., Giner L., Casals N. (2011). Isolation of pluripotent stem cells from human third molar dental pulp. Histol. Histopathol..

[16] Atari M., Gil-Recio C., Fabregat M., Garcia-Fernandez D., Barajas M., Carrasco M.A., Jung H.S., Alfaro F.H., Casals N., Prosper F. (2012). Dental pulp of the third molar: A new source of pluripotent-like stem cells. J. Cell Sci..

[17] Nunez-Toldra R., Martinez-Sarra E., Gil-Recio C., Carrasco M.A., Al Madhoun A., Montori S., Atari M. (2017). Dental pulp pluripotent-like stem cells (DPPSC), a new stem cell population with chromosomal stability and osteogenic capacity for biomaterials evaluation. BMC Cell Biol..

[18] Atari M., Caballe-Serrano J., Gil-Recio C., Giner-Delgado C., Martinez-Sarra E., Garcia-Fernandez D.A., Barajas M., Hernández-Alfaro F., Ferrés-Padró E., Giner-Tarrida L. (2012). The enhancement of osteogenesis through the use of dental pulp pluripotent stem cells in 3D. Bone.

[19] Perard M., Le Clerc J., Watrin T., Meary F., Perez F., Tricot-Doleux S., Pellen-Mussi P. (2013). Spheroid model study comparing the biocompatibility of Biodentine and MTA. J. Mater. Sci. Mater. Med..

[20] Guven E.P., Tasli P.N., Yalvac M.E., Sofiev N., Kayahan M.B., Sahin F. (2013). In vitro comparison of induction capacity and biomineralization ability of mineral trioxide aggregate and a bioceramic root canal sealer. Int. Endod. J..

[21] Zanini M., Sautier J.M., Berdal A., Simon S. (2012). Biodentine induces immortalized murine pulp cell differentiation into odontoblast-like cells and stimulates biomineralization. J. Endod..

[22] Zhao X., He W., Song Z., Tong Z., Li S., Ni L. (2012). Mineral trioxide aggregate promotes odontoblastic differentiation via mitogen-activated protein kinase pathway in human dental pulp stem cells. Mol. Biol. Rep..

[23] Ali H., Al-Yatama M.K., Abu-Farha M., Behbehani K., Al Madhoun A. (2015). Multi-lineage differentiation of human umbilical cord Wharton’s Jelly Mesenchymal Stromal Cells mediates changes in the expression profile of stemness markers. PLoS ONE.

[24] Al Madhoun A., Ali H., AlKandari S., Atizado V.L., Akhter N., Al-Mulla F., Atari M. (2016). Defined three-dimensional culture conditions mediate efficient induction of definitive endoderm lineage from human umbilical cord Wharton’s jelly mesenchymal stem cells. Stem Cell Res. Ther..

[25] Voronova A., Al Madhoun A., Fischer A., Shelton M., Karamboulas C., Skerjanc I.S. (2012). Gli2 and MEF2C activate each other’s expression and function synergistically during cardiomyogenesis in vitro. Nucleic Acids Res..

[26] Al Madhoun A.S., Voronova A., Ryan T., Zakariyah A., McIntire C., Gibson L., Shelton M., Ruel M., Skerjanc I.S. (2013). Testosterone enhances cardiomyogenesis in stem cells and recruits the androgen receptor to the MEF2C and HCN4 genes. J. Mol. Cell. Cardiol..

[27] Nunez-Toldra R., Dosta P., Montori S., Ramos V., Atari M., Borros S. (2017). Improvement of osteogenesis in dental pulp pluripotent-like stem cells by oligopeptide-modified poly(beta-amino ester)s. Acta Biomater..

[28] Hunter A., Archer C.W., Walker P.S., Blunn G.W. (1995). Attachment and proliferation of osteoblasts and fibroblasts on biomaterials for orthopaedic use. Biomaterials.

[29] Yoo E., Paganessi L.A., Alikhan W.A., Paganessi E.A., Hughes F., Fung H.C., Rich E., Seong C.M., Christopherson K.W. (2013). Loss of CD26 protease activity in recipient mice during hematopoietic stem cell transplantation results in improved transplant efficiency. Transfusion.

[30] Torabinejad M., Hong C.U., McDonald F., Pitt Ford T.R. (1995). Physical and chemical properties of a new root-end filling material. J. Endod..

[31] Takita T., Hayashi M., Takeichi O., Ogiso B., Suzuki N., Otsuka K., Ito K. (2006). Effect of mineral trioxide aggregate on proliferation of cultured human dental pulp cells. Int. Endod. J..

[32] Gandolfi M.G., Pagani S., Perut F., Ciapetti G., Baldini N., Mongiorgi R., Prati C. (2008). Innovative silicate-based cements for endodontics: A study of osteoblast-like cell response. J. Biomed. Mater. Res. A.

[33] Attik G.N., Villat C., Hallay F., Pradelle-Plasse N., Bonnet H., Moreau K., Colon P., Grosgogeat B. (2014). In vitro biocompatibility of a dentine substitute cement on human MG63 osteoblasts cells: Biodentine versus MTA(^®^). Int. Endod. J..

[34] Kulterer B., Friedl G., Jandrositz A., Sanchez-Cabo F., Prokesch A., Paar C., Scheideler M., Windhager R., Preisegger K.-H., Zlatko Trajanoski Z. (2007). Gene expression profiling of human mesenchymal stem cells derived from bone marrow during expansion and osteoblast differentiation. BMC Genom..

[35] Hakki S.S., Bozkurt B.S., Ozcopur B., Gandolfi M.G., Prati C., Belli S. (2013). The response of cementoblasts to calcium phosphate resin-based and calcium silicate-based commercial sealers. Int. Endod. J..

[36] Luo Z., Li D., Kohli M.R., Yu Q., Kim S., He W.X. (2014). Effect of Biodentine on the proliferation, migration and adhesion of human dental pulp stem cells. J. Dent..

[37] Ogata H., Hayashi M., Tsuda H., Suzuki N., Maeno M., Sugawara A., Ogiso B. (2012). Effects of a calcium phosphate cement on mineralized nodule formation compared with endodontic cements. Dent. Mater. J..

[38] Peng W., Liu W., Zhai W., Jiang L., Li L., Chang J., Zhu Y. (2011). Effect of tricalcium silicate on the proliferation and odontogenic differentiation of human dental pulp cells. J. Endod..

[39] Dewi A.H., Ana I.D., Wolke J., Jansen J. (2015). Behavior of POP–calcium carbonate hydrogel as bone substitute with controlled release capability: A study in rat. J. Biomed. Mater. Res. Part A.

[40] Simon S., Smith A.J., Lumley P.J., Berdal A., Smith G., Finney S., Cooper P.R. (2009). Molecular characterization of young and mature odontoblasts. Bone.

[41] Eid A.A., Hussein K.A., Niu L.N., Li G.H., Watanabe I., Al-Shabrawey M., Pashley D.H., Tay F.R. (2014). Effects of tricalcium silicate cements on osteogenic differentiation of human bone marrow-derived mesenchymal stem cells in vitro. Acta Biomater..

